# Enhanced Magnetostrain in a <0 0 1>_A_-Textured Ni_44.5_Co_4.9_Mn_37.5_In_13.1_ Alloy through Superelastic Training

**DOI:** 10.3390/ma15062072

**Published:** 2022-03-11

**Authors:** Lanyu Guo, Zongbin Li, Jiaxing Chen, Bo Yang, Haile Yan, Xiang Zhao, Claude Esling, Liang Zuo

**Affiliations:** 1 Key Laboratory for Anisotropy and Texture of Materials (Ministry of Education), School of Materials Science and Engineering, Northeastern University, Shenyang 110819, China; glanyu609@163.com (L.G.); m18804027763@163.com (J.C.); yangb@atm.neu.edu.cn (B.Y.); yanhaile@mail.neu.edu.cn (H.Y.); zhaox@mail.neu.edu.cn (X.Z.); lzuo@mail.neu.edu.cn (L.Z.); 2 Laboratoire d’Étude des Microstructures et de Mécanique des Matériaux (LEM3), CNRS UMR 7239, Université de Lorraine, 57045 Metz, France; claude.esling@univ-lorraine.fr

**Keywords:** meta-magnetic shape memory alloys, magnetostructural transformation, magnetostrain, external field training, texture

## Abstract

Large magnetostrain can be demonstrated in Ni-Mn-X (X = In, Sn, Sb) meta-magnetic shape memory alloys by resuming the predeformed martensite through magnetic-field-induced reverse martensitic transformation. However, owing to the constraint from the self-accommodated microstructure and randomly distributed crystallographic orientation, spontaneous magnetostrain without predeformation in polycrystalline alloys remains low. Here, by combining microstructure texturing and superelastic training, enhanced spontaneous magnetostrain was achieved in a directionally solidified Ni_44.5_Co_4.9_Mn_37.5_In_13.1_ alloy with strong <0 0 1>_A_ preferred orientation. After superelastic training through cyclic compressive loading/unloading on the directionally solidified alloy, a large spontaneous magnetostrain of ~0.65% was obtained by applying a magnetic field of 5 T, showing great improvement when compared to that of the untrained situation, i.e., ~0.45%. Such enhanced magnetoresponse is attributed to the internal stress generated through superelastic training, which affects the variant distribution and the resultant output strain in association with the martensitic transformation.

## 1. Introduction

Heusler-type Ni-Mn-X (X = In, Sn, Sb) intermetallic compounds have received increasing attention in the past two decades, because various functional behaviors linked with the martensitic transformation in these compounds, e.g., magnetic shape memory effect [1,2,3,4,5,6], magnetocaloric effect [7,8,9,10,11,12], elastocaloric effect [13,14,15,16,17,18] and magnetoresistance effect [19,20], have potential applications in areas including actuation, sensing and solid-state refrigeration. These alloys are also referred as meta-magnetic shape memory alloys, with the austenite in a ferromagnetic state and the martensite in a weak magnetic (anti-ferromagnetic or paramagnetic) state [1]. Such coupled changes in magnetic and structural orders thus allow a decrease in the martensitic transformation temperature upon applying a magnetic field. Consequently, the shape change caused by the deformation of martensite can be resumed on account of the reverse martensitic transformation induced by the magnetic field [1], giving rise to a one-way magnetic shape memory effect. Previously, giant magnetostrain up to ~3% has been achieved in a prestrained Ni_45_Co_5_Mn_36.7_In_13.3_ single crystal [1]. Moreover, spontaneous magnetostrain without predeformation can also be achieved by such field-induced transformation [5,6], where the magnitude of magnetostrain depends on the output strain inherent to the martensitic transformation. Notably, when the variation of transformation temperature driven by the magnetic field is wider than the width of thermal hysteresis concomitant with the martensitic transformation [21,22], the field-induced reverse martensitic transformation exhibits reversible behavior on the removal of the magnetic field. In this condition, a reversible or two-way magnetic shape memory effect can be expected.

As the martensitic transformation features a reduction in lattice symmetry, the elastic strain rendered by structural change is a principal barrier for the nucleation and growth of martensite. To offset the elastic strain of individual variants and thus reduce the transformation barrier, the formation of self-accommodated microstructures assembled by various martensite variants is energetically favourable in the absence of external influences. However, due to mutual compensation in elastic strain among variants, the self-accommodated configuration significantly diminishes the output strain in association with the martensitic transformation and the resulting spontaneous magnetostrain. Moreover, although the polycrystalline alloys are easily prepared and thus of low expense, additional constraint from the randomly distributed crystallographic orientation may further weaken the spontaneous magnetostrain. For example, in a polycrystalline Ni_50_Mn_34_In_16_ alloy, the spontaneous magnetostrain is only 0.14% [6], far below the predeformed situation. In this regard, achieving large spontaneous magnetostrain through field-induced reverse martensitic transformation in polycrystalline Ni-Mn-X alloys is still quite challenging.

It is worth mentioning that external field training [23,24,25,26], such as repeated deformation in martensite, thermal cycling under a constant load (stress) during martensitic transformation and superelastic cycling under a constant temperature, can effectively improve the two-way shape memory effect in shape memory alloys. Especially, certain internal stress introduced through external field training favors the formation of preferentially oriented variants upon the martensitic transformation [24,25] and thus results in a substantial increase in the output strain along with the martensitic transformation in the stress-free condition [24]. Inspired by this fact, it is of particular interest to explore the enhanced magnetostrain in Ni-Mn-X meta-magnetic shape memory alloys with no predeformation, by utilizing the internal stress generated through external field training.

In the present work, superelastic training through cyclic isothermal loading/unloading was exploited to enhance the spontaneous magnetostrain in meta-magnetic shape memory alloys. Since the <0 0 1>_A_ orientation can generate the maximum value of transformation strain in Ni-Mn-X alloys [27,28], a <0 0 1>_A_ textured microstructure is conceived to be favorable to increase the potential output strain. Furthermore, the highly textured microstructure can also significantly reduce the internal constraints from the orientation diversity on the lattice deformation in polycrystalline alloys [29]. Here, by taking advantage of the temperature gradient effect of directional solidification, a polycrystalline Ni_44.5_Co_4.9_Mn_37.5_In_13.1_ alloy was prepared consisting of columnar-shaped grains and strong <0 0 1>_A_ texture along the solidification direction (SD). By performing the superelastic training through the cyclic compressive loading/unloading along the SD on the directionally solidified alloy, a large spontaneous magnetostrain of ~0.65% was achieved by the field-induced reverse martensitic transformation under 5 T, being much higher than that of the untrained situation, i.e., ~0.45%.

## 2. Materials and Methods

A button-shaped Ni-Co-Mn-In polycrystalline ingot weighing ~100 g was firstly fabricated by means of arc-melting, using high-purity metals (Boyu Metal Products, Shenyang, China) as the raw materials. To gain strong preferred orientation, directional solidification was performed by using the Bridgman method. The arc-melted alloy was remelted by heating to 1423 K in a corundum crucible with an internal diameter of ~11 mm. After holding for 1 h at 1423 K, the corundum crucible was pulled into the coolant (i.e., liquid metal Ga-In-Sn at room temperature) at a movement rate of 50 μms^−1^, where the temperature gradient was estimated to be ~100 Kcm^−1^. For a homogeneous distribution in composition, the directionally solidified alloy was subject to post annealing at 1173 K in a vacuumed quartz tube. After holding for 24 h, the alloy was quenched in ice water. By means of the Energy Dispersive Spectrometer (EDS, JEOL, Tokyo, Japan) measurements, the composition for the directionally solidified alloy was experimentally determined to be Ni_44.5_Co_4.9_Mn_37.5_In_13.1_ (at %), by averaging the compositions of three regions, as demonstrated in Table 1. For the mechanical treatments and strain measurements, several rectangular samples (6 × 4 × 3 mm) with the longer edge parallel to the SD were cut from the as-homogenized directionally solidified alloy.

The detection of characteristic temperatures for the onset and end of forward/reverse martensitic transformation (*M_s_, M_f_, A_s_, A_f_*) was performed in a differential scanning calorimeter (DSC, TA-DSC25, TA Instruments, New Castle, USA). The temperature-dependent magnetization under a constant magnetic field was recorded in a Quantum Design MPMS-3 system(Quantum Design, San Diego, USA). The crystal structure was analyzed by powder X-ray diffraction (XRD, Rigaku, Tokyo, Japan) with Cu-Kα radiation. The microstructural features were examined using a scanning electron microscope (Jeol JSM-7001F, Tokyo, Japan). The preferred orientation was identified by measuring the incomplete pole figures using XRD on the transverse section (i.e., perpendicular to the SD) of the directionally solidified alloy. The stress–strain curves under compression were monitored in a material testing machine equipped with a temperature control attachment, where the loading direction was along the SD. The magnetostrain was tested in a Quantum Design Physical Property Measurement System (PPMS, Dynacool-9, Quantum Design, San Diego, USA), using the strain-gauge method.

## 3. Results and Discussion

**Figure 1:**
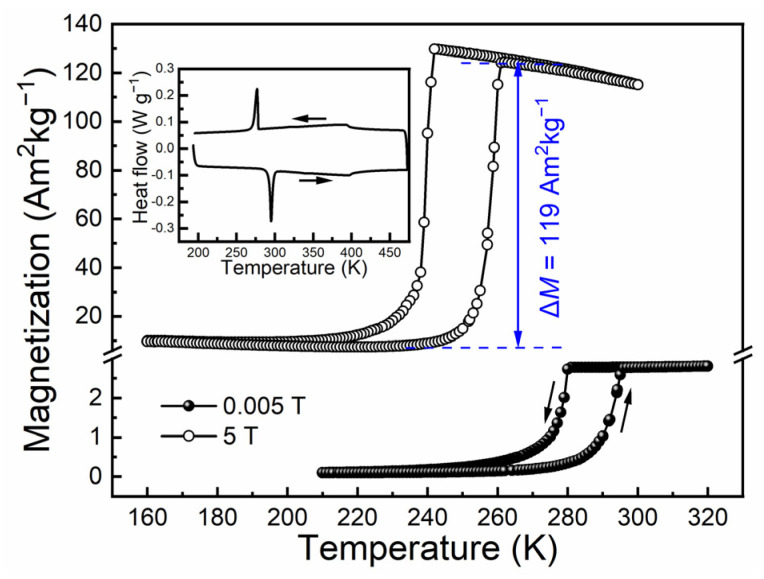
*M* (*T*) curves for the directionally solidified Ni_44.5_Co_4.9_Mn_37.5_In_13.1_ alloy measured under 0.005 T and 5 T. The DSC curves are shown in the inset.

The phase transformation behaviors were analyzed by measuring the temperature dependence of magnetization (*M*(*T*) curves) at 0.005 T and 5 T, as shown in Figure 1. At 0.005 T, it is indicated that the directionally solidified Ni_44.5_Co_4.9_Mn_37.5_In_13.1_ alloy underwent reversible first-order martensitic transformation, where the corresponding transformation temperatures determined by tangent method were *M_s_* = 280 K, *M_f_* = 269 K, *A_s_* = 286 K, and *A_f_* = 297 K. These characteristic temperatures well coincide with those acquired from DSC measurements (inset of Figure 1). At 5 T, it is verified that across the martensitic transformation, there existed a large magnetization difference Δ*M* of 119 Am^2^kg^−1^, which confirms that the reversible martensitic transformation took place between ferromagnetic austenite and weak magnetic martensite. Notably, the martensitic transformation was driven to much lower temperature range when the magnetic field of 5 T is applied, i.e., *M_s_* = 242 K, *M_f_* = 231 K, *A_s_* = 250 K and *A_f_* = 261 K, suggesting that the magnetic field could be employed to drive the reverse martensitic transformation [1]. Owing to the large drop of 36 K in *A_s_* induced by 5 T, the field dependence of transformation temperature shift (i.e., Δ*T*/μ_0_Δ*H*) was as large as 7.2 KT^−1^. This Δ*T*/μ_0_Δ*H* value was close to those in Ni-Co-Mn-In alloys with very similar compositions, i.e., 7.6 KT^−1^ for a Ni_44.9_Co_4.9_Mn_36.9_In_13.3_ alloy [30], 6.7 KT^−1^ for a Ni_45.2_Co_5.1_Mn_36.7_In_13_ alloy [2], and 8.0 KT^−1^ for a Ni_45.7_Co_4.2_Mn_36.6_In_13.5_ alloy [31]. Achieving large Δ*T*/μ_0_Δ*H* value is of great significance to the realization of magnetically driven reverse martensitic transformation and the resulting functional behaviors.

The shift of phase transformation temperatures rendered by the magnetic field can be explained using the Clausius–Clapeyron relation [1], i.e., Δ*T*/μ_0_Δ*H* = Δ*M*/Δ*S_tr_*. According to the Δ*M* value indicated by magnetization measurements and the transformation entropy change Δ*S_tr_* determined by DSC measurements (i.e., 16.7 Jkg^−1^K^−1^) for the present directionally solidified Ni_44_._5_Co_4.9_Mn_37.5_In_13.1_ alloy, the value of Δ*M*/Δ*S_tr_* was calculated to be 7.1 KT^−1^, which was in agreement with the value of 7.2 KT^−1^ shown by the magnetization measurements. Notably, owing to the high Δ*T*/μ_0_Δ*H* value for the present alloy, there existed an obvious gap between the transformation temperature regions at 5 T and 0.005 T, as the *A_f_* under 5 T (i.e., 261 K) was lower than the *M_f_* under 0.005 T (i.e., 269 K). Such effect implies that a field of 5 T can be utilized to drive the reversible field-induced reverse martensitic transformation [21,22], thus giving rise to the reversible magnetoresponse. Especially, within the temperature region bounded by the *A_f_* at 5 T and the *M_f_* at 0.005 T, the reverse transformation from martensite to austenite induced by magnetic field can be complete and reversible [21,22].

**Figure 2:**
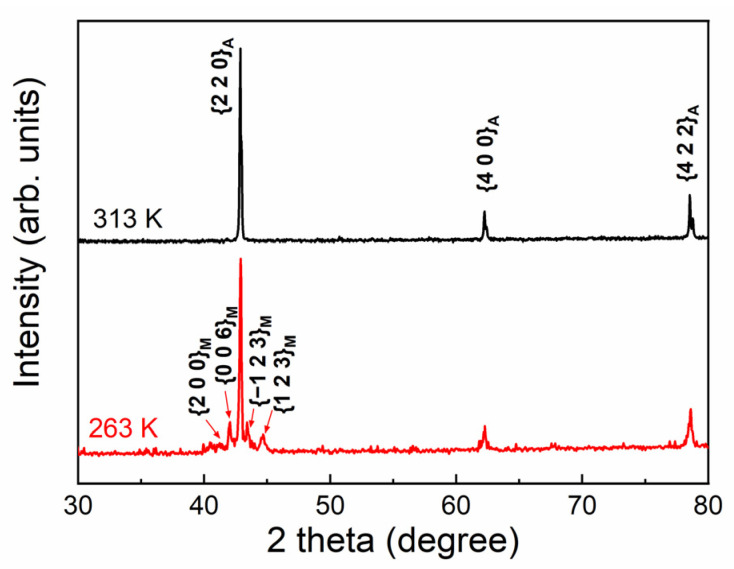
Powder XRD patterns measured at 313 K and 263 K for the directionally solidified Ni_44.5_Co_4.9_Mn_37.5_In_13.1_ alloy.

Figure 2 shows the powder XRD patterns measured at 313 K and 263 K for the directionally solidified Ni_44.5_Co_4.9_Mn_37.5_In_13.1_ alloy. According to the pattern at 313 K, the austenite had a cubic L2_1_ structure with the lattice parameter *a_A_* = 5.995 Å. From the pattern at 263 K, the martensite can be identified as a monoclinic six-layered modulated (6M) structure [28], where the lattice parameters were determined to be *a_M_* = 4.342 Å, *b_M_* = 5.495 Å, *c_M_* = 13.371 Å and *β* = 93.441°. Consequently, the martensitic transformation occurred from cubic austenite to monoclinic 6M martensite.

**Figure 3:**
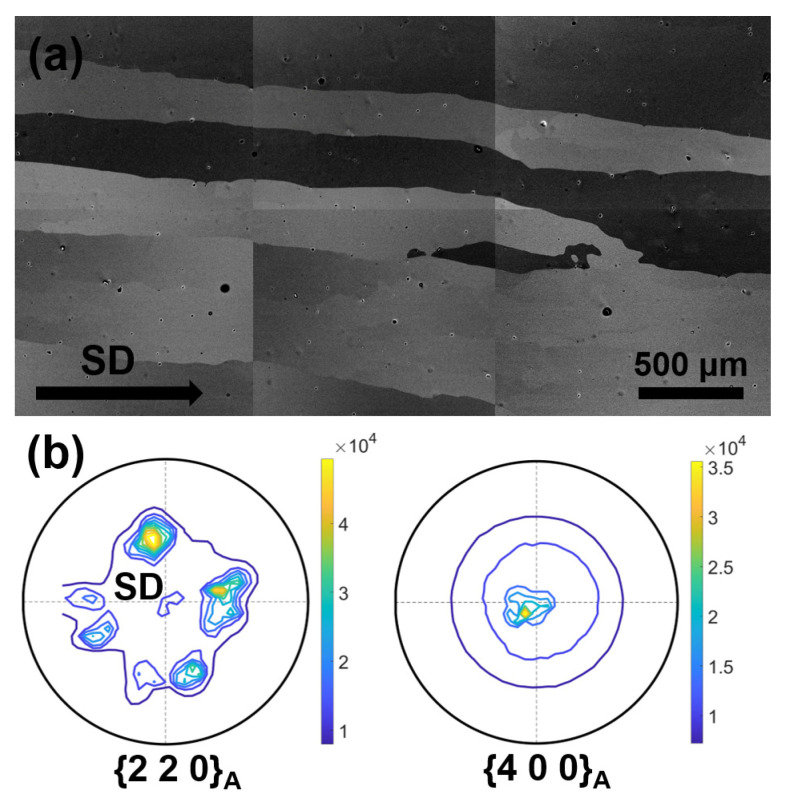
(**a**) Microstructural image of the longitudinal section of the directionally solidified Ni_44.5_Co_4.9_Mn_37.5_In_13.1_ alloy at room temperature. (**b**) {2 2 0}_A_ and {4 0 0}_A_ incomplete pole figures constructed by XRD measurements on the transverse section of the directionally solidified alloy at room temperature. The center of pole figure corresponds to the SD.

Figure 3a displays the microstructure image on the longitudinal section for the directionally solidified Ni_44.5_Co_4.9_Mn_37.5_In_13.1_ alloy, where several micrographs were combined in order to illustrate the microstructural features on an enlarged scale. It shows that the columnar-shaped austenite grains, several hundred microns in size, stretch roughly along the SD. Moreover, as indicated by the {2 2 0}_A_ and {4 0 0}_A_ pole figures of austenite determined from XRD measurements shown in Figure 3b, the austenite also forms a strong preferential orientation with <0 0 1>_A_ parallel to the SD. The formation of the <0 0 1>_A_-textured columnar-shaped grains should be the result of temperature gradient effect in the directional solidification, as the <0 0 1> direction of cubic structured alloys is the favorable growth direction along the temperature gradient. It has been noted that such typical microstructural features may significantly reduce the intergranular constraints on the lattice deformation of martensitic transformation in polycrystalline alloys [29], being favorable for improvement in the transformation strain and the resultant magnetostrain through field-induced magnetostructural transformation.

**Figure 4:**
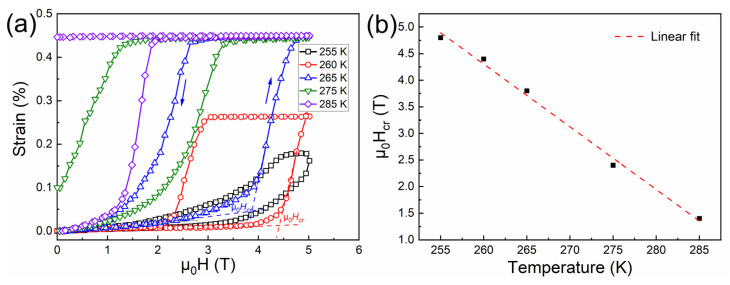
(**a**) Field dependence of strain for the directionally solidified Ni_44.5_Co_4.9_Mn_37.5_In_13.1_ alloy measured at various temperatures when applying a magnetic field up to 5 T. (**b**) Temperature dependence of critical magnetic field (μ_0_*H_cr_*) to drive the reverse martensitic transformation.

Figure 4a plots the field dependence of magnetostrain for the directionally solidified Ni_44.5_Co_4.9_Mn_37.5_In_13.1_ alloy measured under the maximum field up to 5 T at selected temperatures, where the strain values along the SD were measured and the magnetic field was applied perpendicular to the SD. To eliminate the influence of sample history on the magnetostrain measurements, discontinuous heating protocol was employed. Before recording the magnetostrain at each temperature point, the sample temperature was first decreased to 210 K with zero-field to obtain a full martensitic state. Then, the sample was heated to the target temperature point with zero-field, at which the strain values were recorded with the increase and decrease of the magnetic field. It can be seen that the strain recorded at each testing temperature began to increase rapidly when the magnetic field was increased to a certain critical value (μ_0_*H_cr_*), at which the field-induced reverse martensitic transformation was initialized. As demonstrated in Figure 4b, μ_0_*H_cr_* decreases almost linearly with the increase in testing temperature, with a reduction rate of 0.12 TK^−1^.

It can be seen in Figure 4a that the magnetostrain behavior was highly dependent on the testing temperature. At 255 K, a relatively low magnetostrain value of ~0.18% was obtained at 5 T. On raising the testing temperature to 260 K, the magnetostrain was increased to ~0.26%, which should be attributed to the increase in the volume fraction of field-induced transformation as μ_0_*H_cr_* decreases with increasing testing temperature. It is noted that at 255 K and 260 K, the magnetostrain fully recovered to the initial state after removing the magnetic field, but the obtained magnetostrain values were still lower than those at elevated temperatures (i.e., 265 K, 275 K and 285 K). This is because the testing temperatures, i.e., 255 K and 260 K, were just within the temperature interval between the *A_s_* under 5 T (i.e., 250 K) and the *A_f_* under 5 T (i.e., 261 K), where the field-induced reverse martensitic transformation under 5 T was reversible. However, the application of 5 T was not enough to drive a complete transformation. As complete and reversible field-induced transformation behavior occurred in the temperature span between the *A_f_* under 5 T (i.e., 261 K) and the *M_f_* under 0.005 T (i.e., 269 K), a fully reversible magnetostrain of ~0.45% was achieved at 265 K, representing the maximum level of magnetostrain due to the completely reverse transformation. For the temperature region above the *M_f_* under 0.005 T (i.e., 269 K) but below the *M_s_* under 0.005 T (i.e., 280 K), the field of 5 T was capable of driving a complete reverse transformation from martensite to austenite, but the field-induced austenite could not completely transform back to martensite when the field was removed, i.e., partially reversible behavior. Therefore, although large magnetostrain of ~0.45% could also be obtained under a field of 5 T at 275 K, certain residual strain, i.e., 0.1%, could not be recovered on removing the field. With the testing temperature higher than the *M_s_* under 0.005 T, the field-induced transformation was totally irreversible. Thus, the magnetostrain at 285 K remained after removing the field.

**Figure 5:**
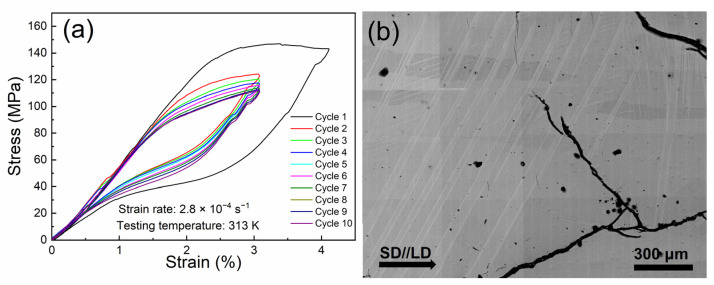
(**a**) Cyclic compressive stress–strain curves of superelastic training for the directionally solidified Ni_44.5_Co_4.9_Mn_37.5_In_13.1_ alloy at 313K with the loading direction (LD) along the SD. (**b**) Microstructure for the alloy after superelastic training at room temperature.

To increase the output strain upon the martensitic transformation and the resultant magnetostrain, cyclic superelastic training was performed on the present alloy. Figure 5a demonstrates the ten cycles of loading/unloading stress–strain curves tested at 313 K (i.e., higher than the *A_f_* point) by applying the compressive loading along the SD. For the first cycle, a compressive strain of 4.2% was applied. Since the compressive stress is applied on the austenite state, the presence of stress plateaus on the stress–strain curves originated from the stress-induced martensitic transformation, which occurred at the critical stress of ~140 MPa. On removing the stress, the compressive strain was able to fully recover through the transformation from martensite to austenite, demonstrating superelastic behavior. For the subsequent loading/unloading cycles, the compressive strain was reduced to 3% in order to prevent potential fracturing. With the increase of superelastic cycles, the critical stress required to initialize the stress-induced martensitic transformation gradually decreased, suggesting the introduction of certain internal stress through superelastic training. At the last two cycles, the critical stress was almost unchanged and remained at the level of ~90 MPa, suggesting the saturation of internal stress. Moreover, microstructural observations show that after superelastic training, some straight martensite plates appeared at the sample surface and they preferred to exhibit an intersection angle of ~60° to the loading direction (i.e., SD), as demonstrated in Figure 5b. The appearance of such preferentially distributed martensite plates could be also an indication that certain internal stress is introduced through superelastic training.

**Figure 6:**
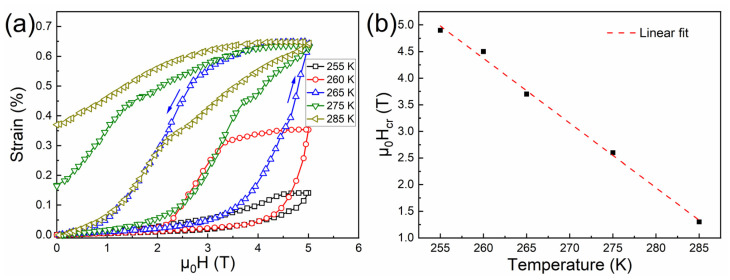
(**a**) Field dependence of magnetostrain for the alloy after superelastic training at selected temperatures with a maximum magnetic field of 5 T. (**b**) Temperature dependence of critical magnetic field (μ_0_*H_cr_*) to drive the reverse martensitic transformation for the alloy after superelastic training.

Figure 6a displays the field dependence of magnetostrain for the alloy after superelastic training at various temperatures with the application of a maximum magnetic field of 5 T. Consistent with the untrained situation, the strain values along the SD were measured by applying the magnetic field in the perpendicular direction. The temperature protocol for strain measurements was the same as that of the untrained situation. In addition, μ_0_*H_cr_* also exhibited a linear reduction when the testing temperature was increased, in agreement with that of the untrained alloy, as shown in Figure 6b.

It can be seen in Figure 6a that at 255 K and 260 K, the reverse martensitic transformation was reversible at 5 T, but was not a complete transformation, giving rise to the corresponding reversible magnetostrain values of ~0.14% and ~0.35% under 5 T, respectively. Enhanced reversible magnetostrain value of ~0.65% was obtained under a field of 5 T at 265 K, where the field-induced reverse martensitic transformation was complete and reversible. At further elevated temperatures, i.e., 275 K and 285 K, the magnetostrain value remains ~0.65% under 5 T. However, there existed certain unrecovered magnetostrain on removing the field. Apparently, compared to that in the untrained alloy (i.e., ~0.45%), the maximum magnetostrain value was significantly improved after superelastic training. Moreover, the present magnetostrain value was also relatively higher than those in certain other Ni-Mn-based alloys described in the literature, as demonstrated in Table 2.

The above results demonstrate that spontaneous magnetostrain can be effectively enhanced through superelastic training in the present directionally solidified Ni_44.5_Co_4.9_Mn_37.5_In_13.1_ alloy. On one hand, the <0 0 1>_A_ textured microstructure can significantly reduce the intergranular constraints on the lattice deformation of martensitic transformation, which favors the improvement of potential magnetostrain. On the other hand, enhanced magnetostrain should result from the influence of internal stress induced through superelastic training. The internal stress promoted the formation of some preferred martensite variants when the alloy was cooled in the absence of external influence, as evidenced in Figure 5b. Thus, when compared to the findings for self-accommodated martensite to austenite, relatively higher output strain along with reverse martensitic transformation was obtained in the alloy after superelastic training, owing to the reformulated variant distribution induced by internal stress. In this condition, the reverse transformation from martensite to austenite driven by the magnetic field may generate more pronounced magnetostrain values.

**Figure 7:**
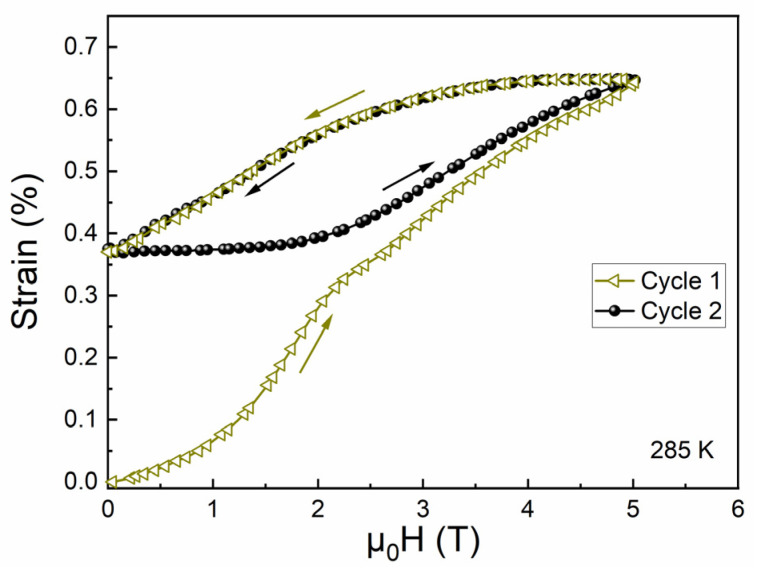
Field dependence of magnetostrain at 285 K for the trained alloy under two cycles of magnetization and demagnetization.

An interesting phenomenon shown in Figure 6 is that certain magnetostrain was recovered (i.e., ~0.28%) when decreasing the magnetic field even at 285 K. This is in sharp contrast to the situation in the untrained alloy, where magnetostrain at 285 K was totally irreversible. To test the reversibility of magnetostrain at 285 K for the alloy after training, an additional cycle of magnetostrain measurements on magnetization and demagnetization was performed, as demonstrated in Figure 7. Consequently, the recovered magnetostrain of ~0.28% was fully reversible for the second cycle of measurements. Such reversible behavior could also be an indication of the existence of internal stress in the trained alloy. For the untrained alloy, the recovery of magnetostrain at 285 K on decreasing the magnetic field was unfavorable due to the lack of driving force. After superelastic training, the introduced internal stress may have served as the driving force to induce certain amounts of magnetic-field-induced austenite to revert back to martensite on removing the magnetic field, resulting in the recovery of certain magnetostrain on removing the magnetic field. The present results indicate that the internal stress introduced by proper external field training could be utilized to improve the reversibility of field-induced reverse martensitic transformation and the corresponding magnetoresponse.

## 4. Conclusions

The magnetostrain in a <0 0 1>_A_-textured Ni_44.5_Co_4.9_Mn_37.5_In_13.1_ alloy prepared by directional solidification was investigated. Through the field-induced reverse martensitic transformation, a large magnetostrain of ~0.45% at 5 T was realized in the directionally solidified alloy. After superelastic training through cyclic isothermal loading/unloading, enhanced magnetostrain up to ~0.65% at 5 T was achieved, which is attributed to the generation of favorable variants induced by the internal stress introduced through superelastic training. In addition, it was shown that the internal stress can be utilized to promote the reversibility of field-induced reverse martensitic transformation, as the internal stress may provide the addition driving stress to enable the transformation of magnetic-field-induced austenite back to martensite. The present work demonstrates that magnetostrain in meta-magnetic shape memory alloys can be effectively manipulated by superelastic training.

## Figures and Tables

**Table 1:** Experimentally determined compositions (at %) by EDS for the directionally solidified alloy.

	Actual Composition (at %)			
	Ni	Co	Mn	In
Region 1	44.4	5.2	37.4	13.1
Region 2	44.5	4.7	37.6	13.2
Region 3	44.6	4.8	37.5	13.1
Averaged	44.5	4.9	37.5	13.1

**Table 2:** Comparison of spontaneous magnetostrain between the present alloy and other Ni-Mn-based alloys reported in the literature.

Alloys	Sample Status	Magnetic Field (T)	Magnetostrain (%)	Ref.
Ni_44.5_Co_4.9_Mn_37.5_In_13.1_	Polycrystal	5	0.65	This work
Ni_46_Cu_4_Mn_38_Sn_12_	Polycrystal	5	0.12	[32]
Ni_50_Mn_34_In_16_	Polycrystal	5	0.14	[6]
Ni_42_Co_8_Mn_39_Sn_11_	Polycrystal	12	0.13	[33]
Ni_45_Co_5_Mn_36_In_13.2_Cu_0.8_	Polycrystal	5	0.24	[34]
Ni_45.2_Mn_36.7_In_13.0_Co_5.1_	Polycrystal	5	0.25	[2]
Ni_49_Co_3_Mn_34_In_14_	Polycrystal	3	0.26	[35]
Ni_45_Co_4.5_Pd_0.5_Mn_37_In_13_	Polycrystal	3	0.30	[36]
Mn_49_Ni_38_Fe_4_Sn_9_	Polycrystal	12	0.30	[37]
Ni_45_Co_5_Mn_37_In_13_	Polycrystal	9	0.40	[5]
Ni_45.7_Co_4.2_Mn_37.3_Sb_12.8_	Polycrystal	7	0.42	[38]
Ni_30_Cu_8_Co_12_Mn_37_Ga_13_	Single crystal	8	0.47	[39]
Ni_50_Mn_33_In_13_Ga_4_	Polycrystal	7	0.49	[40]

## Data Availability

The data presented in this study are available on request from the corresponding author.
